# Assessment of Drought–Heat Dual Stress Tolerance in Woody Plants and Selection of Stress-Tolerant Species

**DOI:** 10.3390/life15081207

**Published:** 2025-07-29

**Authors:** Dong-Jin Park, Seong-Hyeon Yong, Do-Hyun Kim, Kwan-Been Park, Seung-A Cha, Ji-Hyeon Lee, Seon-A Kim, Myung-Suk Choi

**Affiliations:** 1 Department of Variety Examination, National Forest Seed and Variety Center, Chungju 27495, Republic of Korea; djp0903@korea.kr; 2 Division of Forest Biodiversity, Korea National Arboretum, Pocheon 11186, Republic of Korea; ysh1820@korea.kr; 3 Division of Environmental Forest Science, Gyeongsang National University, Jinju 52828, Republic of Korea; piment141@gnu.ac.kr (D.-H.K.); qls4347@gnu.ac.kr (K.-B.P.); chai9514@gnu.ac.kr (S.-A.C.); dnlem54@gnu.ac.kr (J.-H.L.); kasiasun06@gnu.ac.kr (S.-A.K.); 4 Institute of Agriculture of Life Science, Gyeongsang National University, Jinju 52828, Republic of Korea

**Keywords:** sequential drought and heat stress, woody plant tolerance, electrolyte leakage index (ELI), relative water content (RWC), oxidative stress recovery

## Abstract

Sequential drought and heat stress pose a growing threat to forest ecosystems in the context of climate change, yet systematic evaluation methods for woody plants remain limited. This study aimed to develop a comprehensive screening platform for identifying woody plant species tolerant to sequential drought and heat stress among 27 native species growing in Korea. A sequential stress protocol was applied: drought stress for 2 weeks, followed by high-temperature exposure at 45 °C. Physiological indicators, including relative water content (RWC) and electrolyte leakage index (ELI), were used for preliminary screening, supported by phenotypic observations, Evans blue staining for cell death, and DAB staining to assess oxidative stress and recovery ability. The results revealed clear differences among species. *Chamaecyparis obtusa*, *Quercus glauca*, and *Q. myrsinaefolia* exhibited strong tolerance, maintaining high RWC and low ELI values, while *Albizia julibrissin* was highly susceptible, showing severe membrane damage and low survival. DAB staining successfully distinguished tolerance levels based on oxidative recovery. Additional species such as *Camellia sinensis*, *Q. acuta*, *Q. phillyraeoides*, *Q. salicina*, and *Ternstroemia japonica* showed varied responses: *Q. phillyraeoides* demonstrated high tolerance, *T. japonica* showed moderate tolerance, and *Q. salicina* was relatively sensitive. The integrated screening system effectively differentiated tolerant species through multiscale analysis—physiological, cellular, and morphological—demonstrating its robustness and applicability. This study provides a practical and reproducible framework for evaluating sequential drought and heat stress in trees and offers valuable resources for urban forestry, reforestation, and climate-resilient species selection.

## 1. Introduction

Global warming, primarily driven by anthropogenic greenhouse gas emissions, has led to increasingly frequent and severe occurrences of drought and heat stress worldwide, resulting in heightened risks to forest ecosystems [1,2,3]. These stress factors exert compound effects on plant physiological functions, often exceeding thresholds that plants can tolerate, thereby leading to widespread declines in productivity, growth, and ultimately increased tree mortality rates [4]. While each of these stressors alone can adversely impact plant health, their sequential occurrence, referred to as sequential drought and heat stress, amplifies physiological disruptions beyond the sum of individual stress factors, making it a critical concern for forest sustainability [5,6,7].

Individual studies on either drought or heat stress have demonstrated that each stress factor triggers distinct physiological responses. Drought stress reduces soil water availability, leading to stomatal closure, decreased transpiration rates, and suppressed photosynthesis, ultimately reducing carbon assimilation and biomass accumulation [8]. Conversely, heat stress damages photosynthetic machinery, particularly affecting Photosystem II efficiency, while also compromising cell membrane integrity and increasing electrolyte leakage, resulting in cellular instability [9]. When both stressors co-occur, their effects are not simply additive but often synergistic, leading to rapid accumulation of reactive oxygen species (ROS), oxidative stress, and cellular dysfunction [5,7,10]. Recent studies emphasize that tree mortality under climate change is often driven not by a single factor but by the combined or sequential effects of drought and heat, which can act synergistically to disrupt plant hydraulic function and carbon metabolism [11]. This highlights the importance of evaluating plant responses under sequential stress conditions rather than in isolation. Therefore, understanding these interactive mechanisms is crucial for developing strategies to enhance plant resilience to climate change.

Plants have evolved diverse physiological strategies to cope with abiotic stresses such as drought, heat, and salinity. These adaptive mechanisms include stress-responsive signaling pathways (e.g., ABA and ROS networks), morphological changes (e.g., stomatal regulation and root structural changes), and epigenetic modifications that enhance tolerance across generations [12,13]. Therefore, the analysis of signaling and antioxidant compounds, along with the observation of structural alterations, may serve as effective indicators for evaluating plant sensitivity or resistance to abiotic stress.

Although substantial research has been conducted on crop species to screen and breed genotypes with improved tolerance to drought and heat, studies on woody plants, which play vital ecological and economic roles, remain notably underexplored. In woody plants, drought mainly triggers stomatal closure and osmotic regulation, while heat stress compromises membrane integrity and causes protein denaturation, both negatively affecting growth and photosynthesis [14,15]. When combined, these stresses amplify one another, resulting in more pronounced reductions in carbon balance and redox homeostasis compared to individual stress conditions [16,17]. Gaining insight into these interactive responses is critical for strengthening the adaptive capacity of woody species.

Previous studies highlighted significant losses in both agricultural and forest ecosystems due to combined drought and heat stress. For example, from 1980 to 2012, economic losses in U.S. agriculture caused by drought–heat stress were estimated at USD 200 billion—four times higher than losses from drought alone [10]. Furthermore, intensifying drought–heat stress events are projected to accelerate tree mortality worldwide, with cases already documented in North America, Europe, and Australia [1,4]. Despite these alarming trends, research focusing explicitly on drought–heat dual stress tolerance in forest tree species is lacking, particularly in terms of physiological assessments and effective screening methodologies.

Several studies on agricultural crops, such as wheat, chickpea, and groundnut, have successfully identified stress-tolerant genotypes by analyzing key physiological traits under combined drought and heat stress conditions [18,19,20]. In contrast, comparable efforts for woody plants are scarce and have largely focused on single stress conditions rather than sequential stress responses [21,22]. Woody plants have complex and prolonged life cycles, requiring more sophisticated water regulation mechanisms, making their responses to sequential stress conditions both challenging to assess and highly variable [2]. For example, Correia et al. [23] reported that *Eucalyptus globulus* exhibited unique protective responses under sequential drought and heat stress that were not activated by either stress alone, highlighting the importance of examining stress combinations. This gap underscores the urgent need for systematic research on sequential drought and heat stress tolerance in trees to enhance forest resilience and adaptation strategies under climate change.

The evaluation of plant responses to combined drought and heat stress, as well as the identification of tolerant species, has predominantly been conducted under field conditions [19,20,24]. This approach is suitable for crops, which are typically cultivated in the field and allow for large-scale experimentation. Moreover, field-grown plants experience natural drought and heat stress, directly reflecting the impacts of climate change. However, field conditions are inherently variable compared to controlled laboratory settings, meaning tolerance is often assessed in relative terms. Plant stress tolerance may also fluctuate from year to year due to environmental inconsistency. To address these limitations, some researchers have evaluated combined drought and heat stress under controlled laboratory conditions [6,7,24,25]. In these settings, morphological and physiological changes in plants exposed to combined stress were distinguishable from those subjected to single drought or heat stress. This controlled approach is reliable, allows for precise manipulation of stress parameters, and is particularly suited for woody plants, which require extended timeframes for accurate stress tolerance assessment.

This study is based on the hypothesis that physiological analyses can serve as effective tools for evaluating plant responses to combined drought and heat stress, and for distinguishing between tolerant and sensitive woody species. Accordingly, the main objective of this research was to develop and validate a screening framework to assess stress tolerance in native Korean woody plants. Key physiological indicators included relative water content (RWC) to evaluate drought tolerance and the electrolyte leakage index (ELI) to assess heat tolerance. In addition, Evans blue staining was used to examine cell membrane damage, and 3,3′-diaminobenzidine (DAB) staining was applied to evaluate oxidative stress recovery, enabling a comprehensive assessment at the cellular level. Through this multifaceted approach, the study aimed to elucidate species-specific responses under sequential drought and heat stress and to identify tree species with superior tolerance. The findings are expected to provide a valuable foundation for breeding programs, conservation strategies, and afforestation initiatives that address the impacts of climate change on forest ecosystems.

## 2. Materials and Methods

### 2.1. Plant Materials

The study was conducted to screen and identify sequential drought and heat stress-tolerant woody plants among 27 woody species growing in Korea, which were evaluated under controlled environmental conditions. The list of plant species used in this study is presented in Table 1. Seeds of each plant were provided by Gyeongsangnam-do Forest Environment Research Institute (Jinju, Republic of Korea) and the Korean National Arboretum (Pocheon, Republic of Korea), which were collected from natural habitats in 2021. They were germinated in the experimental forest at Gyeongsang National University, and one-year-old seedlings of each species were obtained. To minimize experimental errors due to inter-individual variation, three individuals with similar growth patterns were selected for each plant species and used in the experiment.

### 2.2. Drought–Heat Dual Stress Treatment

In this study, a modified version of the method described by Rizhsky et al. [7] was applied to evaluate sequential drought and heat stress tolerance in woody plants. Rizhsky et al. [7] induced heat stress by increasing the temperature in a growth chamber and imposed drought stress by withholding water until the plants reached a relative water content of 70% to 75%. The sequential drought and heat stress were applied sequentially, first by subjecting plants to drought stress, followed by heat stress treatment. In this context, the term “sequential” refers to the intentional order of stress application, in which drought stress is followed by heat stress, and it does not imply multiple phases of drought. This sequential treatment was intended to reflect actual climate conditions, and the drought stress applied was of sufficient intensity to induce physiological stress rather than serving as a mild pre-treatment to enhance tolerance. In this study, while following the original process described by Rhizsky et al. [7], the drought and heat stress conditions were modified to account for the climate conditions in Korea and the characteristics of the plant species used.

Seeds of the woody plant species used in this study were collected in the fall of 2021 and sown in a greenhouse in March 2022. From May 2022, the seedlings were grown outdoors for one year. In April 2023, prior to stress treatment, the one-year-old seedlings were transplanted into 32-cell plastic pot trays with an upper diameter of 47 mm, a lower diameter of 27 mm, a depth of 96 mm, and a volume of 110 cm^3^. The trays were made of polypropylene and equipped with drainage holes to prevent waterlogging. Transplanting was conducted to provide uniform container conditions before stress treatment, to standardize the growing environment, and to facilitate consistent management and handling during the experimental period. The roots were carefully transferred with the original soil intact to minimize root disturbance and transplanting stress.

The substrate used was a commercial peat moss-based bed soil mixed with perlite at a volume ratio of 2:1. No additional fertilization was applied during the nursery or experimental periods. All seedlings were grown under natural light conditions without supplemental lighting.

Stress treatment was conducted in May 2023, at which point the seedlings had fully expanded leaves and had entered an active vegetative growth phase beyond the early developmental stage, ensuring mature and consistent physiological responses. After a 10-day acclimatization period following transplantation, sequential drought and heat stresses were applied. Drought stress was induced by withholding irrigation for two weeks, followed by a six-hour exposure to high-temperature conditions (40 °C).

During the stress period, relative humidity was maintained at 45–55%, and all plants were kept under the same greenhouse conditions. The appropriateness of the two-week drought treatment was confirmed through a preliminary trial, in which soil water potential dropped below −2.5 MPa, a level known to induce significant physiological stress in plants.

Following drought stress, high-temperature stress was applied by placing the drought-treated plants in a growth chamber set to 45 °C for 120 min. The temperature and exposure duration were selected to mimic extreme heat conditions that may occur in natural environments, focusing on evaluating cell membrane stability, water loss rate, and oxidative stress responses.

**Figure 1:**
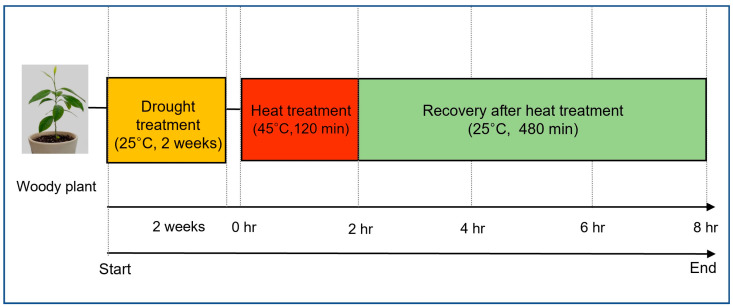
Schemes of drought–heat dual stress treatments. This scheme was designed by modifying the method in the report of Rizhsky et al. [7].

After the sequential drought and heat stress treatment, the plants were transferred to a growth chamber maintained at 25 °C for recovery. Plants were rewatered before being transferred to the recovery phase to ensure that all individuals entered the recovery stage under the same soil moisture condition, allowing a more accurate comparison of recovery ability across species. During this phase, physiological responses were continuously monitored to assess the recovery ability and survival rate of each species after being exposed to combined stress conditions. An overview of the sequential drought and heat stress treatment process is presented in Figure 1.

### 2.3. Pre-Screening of Drought–Heat Dual Stress Tolerant Plant Species by Measuring ELI and RWC

Electrolyte leakage index (ELI) was measured to pre-screen heat-tolerant plants and to assess cell membrane stability under sequential drought and heat stress conditions. Five leaf disks (Ø 5 mm) were collected from the top 2–4 leaves of each woody plant species, which had not been previously subjected to stress treatment. The leaf disks were placed in 5 mL deionized water inside conical tubes and subjected to heat stress conditions of 45 °C for 60 min. Positive and negative controls were included in the experiment, with the positive control consisting of leaf disks autoclaved at 121 °C for 15 min, and the negative control consisting of leaf disks maintained at 25 °C for 120 min. After heat exposure, all tubes were placed on a shaking incubator (100 rpm, 30 min). The amount of electrical conductivity was determined using a conductivity probe (VE 4810, Korea Scientifics, Seoul, Republic of Korea). Electrolyte leakage index (ELI) was calculated by Formulas (1) and (2):

(1) $$\mathrm{ELI}=\frac{{\mathrm{EL}}_{H}-\;{\mathrm{EL}}_{C}}{{\mathrm{EL}}_{C}}$$

(2) $$\mathrm{Electrolyte}\;\mathrm{leakage}\;\left(\mathrm{EL}\right)=\frac{\mathrm{electrical}\;\mathrm{conductivity}\;\mathrm{in}\;\mathrm{treatment}}{\mathrm{electrical}\;\mathrm{conductivity}\;\mathrm{in}\;\mathrm{positive}\;\mathrm{control}}$$

where EL_H_ represents electrolyte leakage under heat stress conditions and EL_C_ represents electrolyte leakage under negative control conditions.

To evaluate drought tolerance, relative water content (RWC) was measured after dehydration shock. To induce dehydration shock, fully hydrated leaves were detached, and fresh weight changes were monitored for six hours using an electronic balance. Formula (3) was used to calculate RWC:

(3) $$\mathrm{RWC}=\frac{\mathrm{Fresh}\;\mathrm{weight}\;-\mathrm{Dry}\;\mathrm{Weight}}{\mathrm{Turgid}\;\mathrm{weight}\;-\mathrm{Dry}\;\mathrm{Weight}}\times \;100$$

The preliminary selection of drought and heat stress-tolerant species was conducted using a scatter plot visualization based on previously measured RWC and ELI values. Species with low ELI values and high RWC values were pre-selected as sequential drought and heat stress-tolerant candidates, followed by physiological experiments to confirm their tolerance.

### 2.4. Investigation of Cell Dead Tissue by Evans Blue Staining Assay

Leaf samples (1 cm^2^) prepared from each selected plant species, incubated in each normal condition or heat treatment, were incubated with Evans blue solution (1%) for one hour at room temperature. As the positive controls, leaf samples collected from each plant species were boiled in water for 1 h and stained under the same conditions described above. Then, they were extensively washed with distilled water to remove unbound Evans blue dye. Photographs of the leaf samples were taken under the microscope (BH-2, Olympus, Japan) at 200× magnification. To assess dead cells, the stained dye in leaves was dissolved using 2 mL of 50% ethanol solution containing 2% sodium dodecyl sulfate (SDS). The absorbance values of dissolved dye from the leaves were measured at 500 nm. The inhibition indexes were obtained by the following Formula (4):

(4) $$Inhibition\;index=\left(\frac{{\mathrm{Abs}}_{H}\;-{\mathrm{Abs}}_{\mathrm{NC}}}{{\mathrm{Abs}}_{\mathrm{PC}}\;-{\mathrm{Abs}}_{\mathrm{NC}}}\right)\times 100$$

where Abs_H_ represents the absorbance of heat-stressed samples, Abs_NC_ represents the absorbance of negative control, and Abs_PC_ represents the absorbance of positive control.

### 2.5. Investigation of Recovery Ability by DAB Staining Assay

To assess the plant’s ability to recover from oxidative stress, 3,3′-diaminobenzidine (DAB) staining was conducted following the protocol by Daudi and O’Brien [26]. Leaf samples of each selected plant species were collected under normal, drought, and sequential drought and heat stress conditions. Additionally, leaf samples that were infiltrated with 30% (*v*/*v*) hydrogen peroxide were prepared as a positive control. The samples were infiltrated with freshly prepared DAB solution under vacuum for 10 min, followed by incubation on a shaker at 100 rpm for 4 h. After incubation, the DAB solution was replaced with a bleaching solution (ethanol:acetic acid:glycerol = 3:1:1), and then leaves were boiled at 95 °C for 30 min to remove chlorophyll. The stained leaves were observed under a microscope at 200× magnification.

Image quantification was performed based on an analysis protocol using Fiji (ImageJ 1.53, NIH, Bethesda, MD, USA), with modifications adapted to the objectives of this study based on the ROS quantification algorithm proposed by Sekulska-Nalewajko et al. [27]. High-resolution TIFF images (2048 × 2048 px, 24-bit RGB) of DAB-stained leaf tissues were imported into Fiji, and the RGB channels were separated using the RGB Stack function. The red channel, which clearly reflects the DAB reaction, was selected and converted to 8-bit grayscale. Since darker regions represent higher staining intensity due to the nature of DAB accumulation, the contrast was adjusted using the Invert function when necessary. Regions of interest (ROIs) were defined as three fixed square areas (100 × 100 pixels) within each leaf segment, selected from zones showing the most prominent staining. ROIs were consistently applied across species and treatments to ensure comparability. Before saturation measurement, grayscale values from non-stained background areas were extracted from each image and subtracted from the ROI average values to obtain background-corrected saturation values. Final saturation levels were calculated based on the Saturation component of HSV-converted images. The mean and standard deviation (mean ± SD) were calculated from three biological replicates per sample (*n* = 3). All quantitative data were organized and used for statistical analysis using Microsoft Excel and GraphPad Prism 9.

### 2.6. Statistical Analysis

All experiments were conducted using three independent replicates, and data are presented as mean ± standard deviation (SD). Statistical analysis was performed using SPSS software (version 27, IBM, Armonk, NY, USA), with a one-way analysis of variance (ANOVA) followed by Duncan’s multiple range test (DMRT) at a significance level of 0.05

## 3. Results

### 3.1. Pre-Screening Candidates of Tolerant Woody Plants Against Sequential Drought and Heat Stress

Preliminary screening was conducted on four woody plant species (*C. obtusa*, *Q. glauca*, *Q. myrsinaefolia*, and *A. julibrissin*) under sequential drought and heat stress conditions. Drought stress was induced by withholding water for two weeks, followed by exposure to high-temperature stress at 45 °C for 120 min. Following two weeks of sequential drought and heat stress, survival rates varied among species: *C. obtusa*, *Q. glauca*, and *Q. myrsinaefolia* maintained 100% survival, whereas *A. julibrissin* exhibited a significantly lower survival rate of 8.3%. These differences were further confirmed by Evans blue staining, which visually indicated higher levels of cellular damage in *A. julibrissin* compared to the tolerant species.

**Figure 2:**
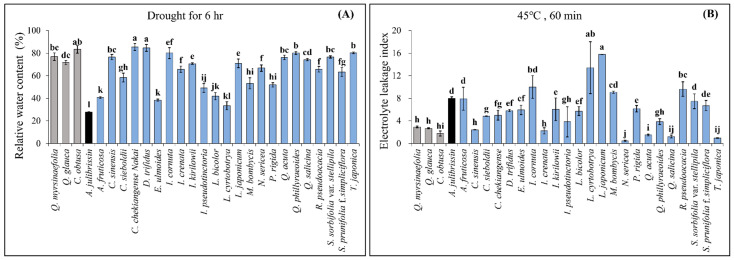
Pre-screening of drought–heat dual stress-tolerant woody plants. (**A**) Identification of drought-tolerant species based on RWC measurements. (**B**) Identification of heat-tolerant species based on ELI measurements. The species with a gray bar represent tolerant species, those with a black bar represent vulnerable species, and those with blue bar are screened species. The data are presented as mean values with standard deviation (SD) from three independent replicates (*n* = 3). Different letters (a–l) indicate statistically significant differences according to Duncan’s multiple range test at the *p* < 0.05 level.

After monitoring the fresh weight of detached leaves for six hours, RWC was measured in 27 woody plant species (Figure 2). Among them, 10 species exhibited RWC values statistically similar to or higher than those of the previously identified tolerant species (*C. obtusa*: 78.2%, *Q. myrsinaefolia*: 76.8%, *Q. glauca*: 72.9%). These species included *C. chekiangense*, *C. sinensis*, *D. trifidus*, *I. kirilowii*, *I. japoncium*, *Q. acuta*, *Q. phillyraeoides*, *Q. salicina*, *S. sorbifolia* var. *stellipila*, and *T. japonica*. In contrast, *A. julibrissin* showed significantly lower RWC (35.6%), indicating its high sensitivity to drought stress.

For heat stress assessment, ELI was measured following exposure to 45 °C for 60 min (Figure 2). Among the 27 tested species, 7 exhibited significantly lower ELI values, comparable to or lower than those of the previously identified tolerant species (*C. obtusa*: 1.78, *Q. myrsinaefolia*: 2.94, *Q. glauca*: 2.68). These species included *I. crenata*, *N. sericea*, *Q. acuta*, *Q. phillyraeoides*, *Q. salicina*, and *T. japonica*. In contrast, *A. julibrissin* showed a high ELI value of 8.00, confirming its sensitivity to heat stress.

**Figure 3:**
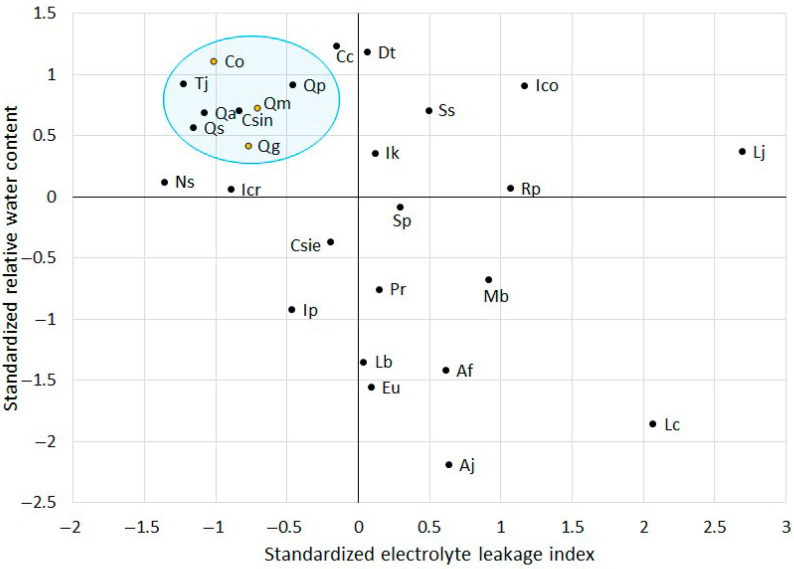
Scatter plot of standardized relative water content and electrolyte leakage index across tree species. The abbreviated labels inside the plot represent scientific names, as listed in Table 1. The orange-dotted species are previously validated heat–drought dual stress-tolerant species. Species surrounded by a blue circle were selected as heat–drought dual stress-tolerant species.

To identify additional stress-tolerant species, the standardized values of RWC and ELI were visualized in a scatter plot (Figure 3). Previously validated stress-tolerant species (*C. obtusa*, *Q. glauca*, and *Q. myrsinaefolia*) were found in quadrant II, which is characterized by high standardized RWC (>0) and low standardized ELI (<0). In contrast, *A. julibrissin* was found in quadrant IV, which is characterized by low RWC and high ELI, indicating high susceptibility to sequential drought and heat stress conditions. This finding aligns with earlier phenotypic observations and histochemical staining results.

Upon analyzing the distribution of the remaining species, five additional species (*C. sinensis*, *Q. acuta*, *Q. phillyraeoides*, *Q. salicina*, and *T. japonica*) were located in close proximity to those three tolerant species within the scatter plot. Their clustering suggests a similar physiological response to environmental stress, specifically maintaining higher water retention and lower electrolyte leakage, traits known to indicate tolerance to drought and heat stress. Given their proximity to previously validated stress-tolerant species, these five species were selected for further experimental assessment to confirm their resilience under additional stress conditions.

### 3.2. Phenotypic Responses of Candidates of Tolerant Woody Plants Against Sequential Drought and Heat Stress

**Figure 4:**
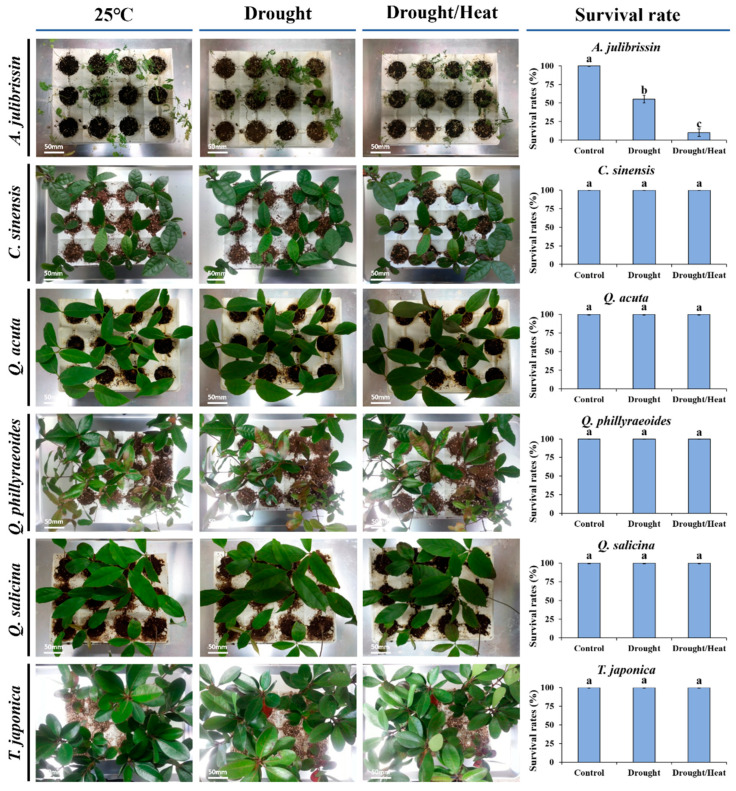
Phenotypic response of the tolerant candidates under drought–heat dual stress. The data are presented as mean values with standard deviation (SD) from three independent replicates (*n* = 3). Different letters (a, b, c) indicate statistically significant differences according to Duncan’s multiple range test at the *p* < 0.05 level.

Figure 4 presents the phenotypic characteristics of previously selected sequential drought and heat stress-tolerant woody plant species under experimental stress conditions. These species, including *C. sinensis*, *Q. acuta*, *Q. phillyraeoides*, *Q. salicina*, and *T. japonica*, were subjected to two weeks of drought stress followed by high-temperature exposure at 45 °C.

The tolerant species exhibited distinct visual indicators of stress resistance. *Q. acuta*, *Q. phillyraeoides*, and *Q. salicina* maintained predominantly green leaves with minimal rolling and exhibited no visible signs of wilting or necrosis. These observations indicate that these species possess stable phenotypic traits under sequential drought and heat stress conditions. *C. sinensis* and *T. japonica* also exhibited relatively stable phenotypic responses, with only slight leaf shrinkage, suggesting strong adaptability to stress.

In contrast, *A. julibrissin*, a known sensitive species, displayed severe stress symptoms, including significant wilting, leaf discoloration, and rapid necrosis, particularly at leaf edges and tips. This stark contrast in phenotypic responses between the tolerant and sensitive species supports the earlier physiological assessments of sequential drought and heat stress tolerance.

These findings confirm that the five candidate species (*C. sinensis*, *Q. acuta*, *Q. phillyraeoides*, *Q. salicina*, and *T. japonica*), which were previously selected based on RWC and ELI, exhibit phenotypic resilience under sequential drought and heat stress conditions. The consistency between physiological and phenotypic indicators provides a strong foundation for further biochemical analyses in these species.

### 3.3. Cellular Damage Assessment of Sequential Drought and Heat Stress-Tolerant Woody Plants Using Evans Blue Staining

**Figure 5:**
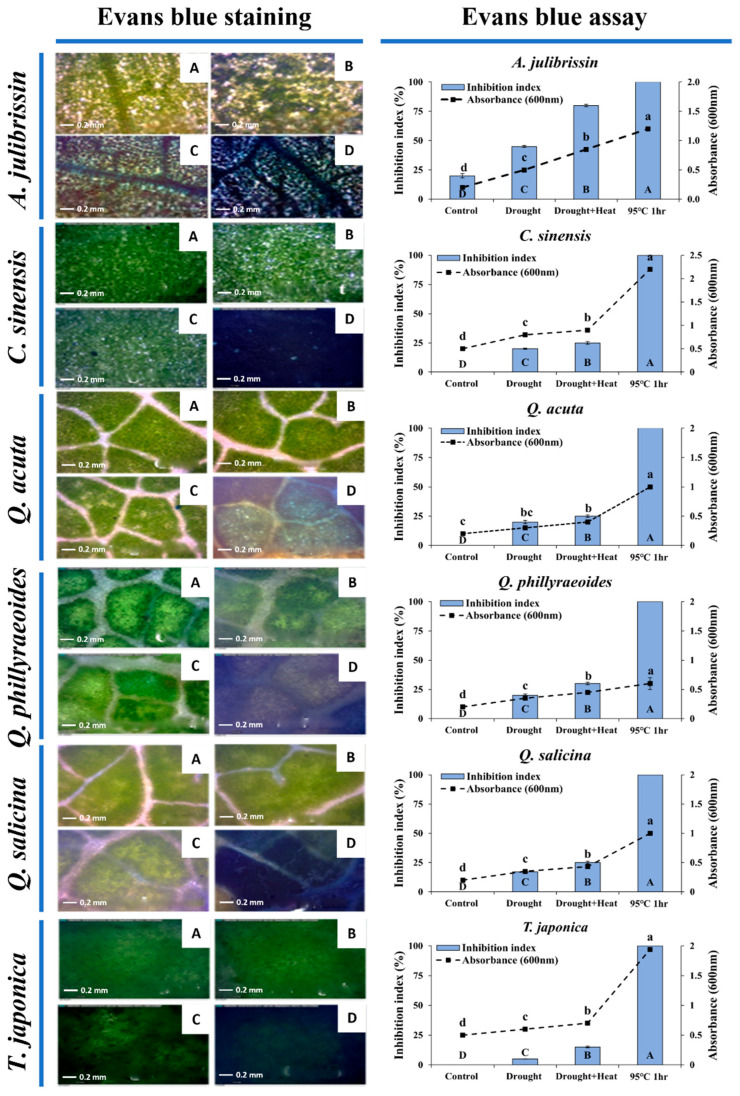
Assessing the tolerances of the drought–heat stress-tolerant woody plants by observing damage using the Evans blue assay. (**A**) control. (**B**) drought treatment. (**C**) drought + heat treatment. (**D**) combined drought and heat treatment at 95 °C for 1 h. One-year-old seedlings, which were exposed to a high temperature after drought stress for two weeks, followed by heat stress at 45 °C for 2 h, were stained with 1% Evans blue dye solution at room temperature for 12 h. The data are presented as mean values with standard deviation (SD) from three independent replicates (*n* = 3). Different letters (a, b, c, d) indicate statistically significant differences according to Duncan’s multiple range test at the *p* < 0.05 level.

Figure 5 presents the results of assessing cellular-level tolerance to sequential drought and heat stress in previously selected woody plant species using Evans blue staining. One-year-old seedlings of the selected tolerant candidates (*C. sinensis*, *Q. acuta*, *Q. phillyraeoides*, *Q. salicina*, and *T. japonica*) and a sensitive control species (*A. julibrissin*) were exposed to drought stress for two weeks, followed by heat stress exposure at 45 °C. Subsequently, leaves from these seedlings were stained with a 1% Evans blue solution at room temperature for 12 h to visually assess the degree of cellular damage.

Results clearly indicated differences in cellular damage among species under sequential drought and heat stress conditions. Leaf tissues of the tolerant candidate species (*C. sinensis*, *Q. acuta*, *Q. phillyraeoides*, and *Q. salicina*) showed minimal staining by Evans blue following both drought stress and sequential drought and heat stress (45 °C), with cellular damage below 30%. *T. japonica* exhibited slight staining in some leaf tissues, but overall cellular damage remained under 15%, indicating strong cellular-level stress tolerance.

In contrast, leaves of the sensitive control, *A. julibrissin*, were heavily stained by Evans blue, with approximately 45% staining after drought stress, increasing to over 80% after sequential drought and heat stress. These findings demonstrate severe cell membrane damage in *A. julibrissin* under sequential drought and heat stress, consistent with earlier phenotypic observations.

In conclusion, Evans blue staining confirmed that sequential drought and heat stress-tolerant candidate species (*C. sinensis*, *Q. acuta*, *Q. phillyraeoides*, *Q. salicina*, and *T. japonica*) possess clear cellular-level tolerance.

### 3.4. Evaluation of Cell Membrane Stability in Sequential Drought and Heat Stress-Tolerant Woody Plants Using Electrolyte Leakage Index

**Figure 6:**
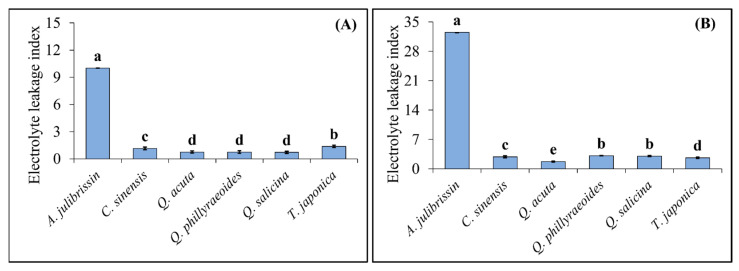
Assessing tolerances of the drought–heat stress-tolerant woody plants by measuring electrolyte leakage indexes after two weeks of drought stress (**A**) and two hours of heat stress at 45 °C following two weeks of drought stress (**B**). The data are presented as mean values with standard deviation (SD) from three independent replicates (*n* = 3). Different letters (a–e) indicate statistically significant differences according to Duncan’s multiple range test at the *p* < 0.05 level.

Figure 6 presents the results of measuring the ELI to evaluate the tolerance of sequential drought and heat stress-tolerant woody plants. One-year-old seedlings of the selected tolerant candidate species (*C. sinensis*, *Q. acuta*, *Q. phillyraeoides*, *Q. salicina*, and *T. japonica*) and the sensitive control species (*A. julibrissin*) were subjected to drought stress for two weeks, followed by exposure to high-temperature conditions at 45 °C. After the treatments, the ELI was measured in leaf tissues to assess the extent of cell membrane damage.

The analysis revealed significant differences in ELI among species under sequential drought and heat stress conditions. Among the tolerant candidate species, *Q. acuta*, *Q. phillyraeoides*, and *Q. salicina* exhibited relatively low ELI values of 0.75, 0.75, and 0.71, respectively, after drought stress. After heat stress following drought stress, their ELI values increased slightly to 1.75, 3.13, and 3.06, respectively, but remained at relatively low levels. Similarly, *T. japonica* showed ELI values of 1.38 and 2.63 after drought stress and sequential drought and heat stress, respectively, demonstrating relatively high membrane stability.

In contrast, the sensitive control species, *A. julibrissin*, exhibited a high ELI of 10.0 after drought stress, which further increased sharply to 32.5 after sequential drought and heat stress, indicating severe cell membrane damage.

### 3.5. Plant Recovery Ability of Selected Woody Plants Under Sequential Drought and Heat Stress

**Figure 7:**
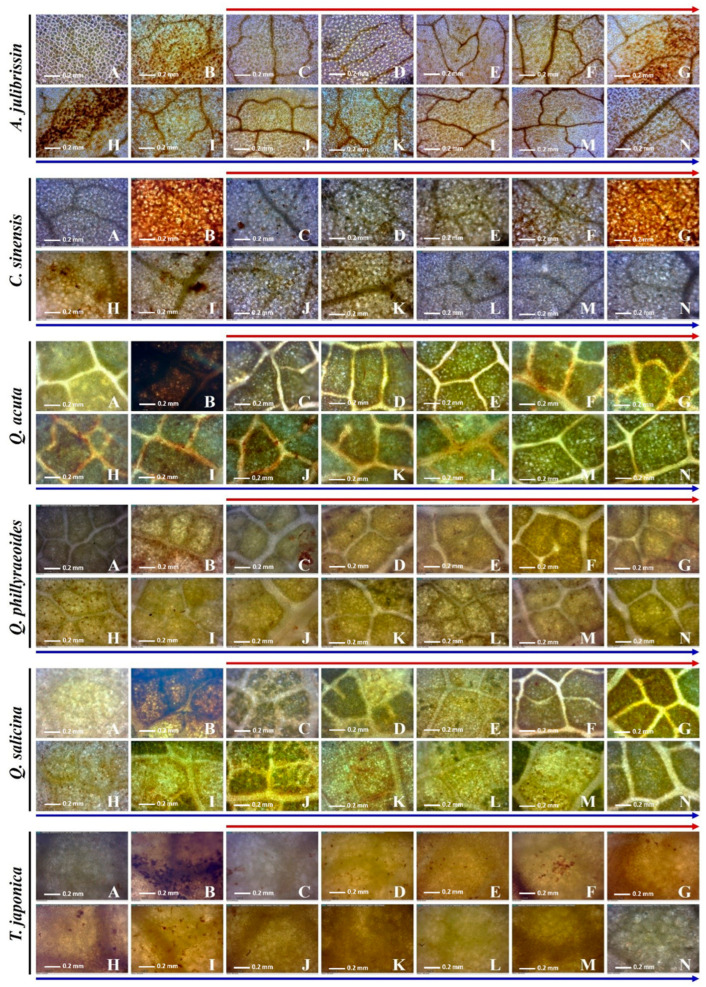
Time-dependent analysis of in situ hydrogen peroxide (H_2_O_2_) accumulation after exposed to two weeks of drought stress (**C**) and drought–heat dual stress at 45 °C (**D**–**G**) and in situ H_2_O_2_ reduction after recovery at 25 °C after heat treatment (**H**–**N**) using DAB staining in drought–heat stress-tolerant woody plants. (**A**) 25 °C after heat stress. (**B**) H_2_O_2_ treatment. (**C**) drought treatment. (**D**) 45 °C, 30 min. (**E**) 45 °C, 60 min. (**F**) 45 °C, 90 min. (**G**) 45 °C, 120 min. (**H**) recovery, 15 min. (**I**) recovery, 30 min. (**J**) recovery, 60 min. (**K**) recovery, 90 min. (**L**) recovery, 120 min. (**M**) recovery, 240 min. (**N**) recovery, 480 min. The red line represents the time elapsed after applying drought and heat stress, while the blue line indicates the recovery period after transferring the plants to 25 °C following heat treatment.

Using DAB staining methods, in situ hydrogen peroxide induced by sequential drought and heat stress was detected (Figure 7). The in situ hydrogen peroxide was visualized by brown precipitate. The leaves of the tolerant and sensitive woody plants were collected under drought and sequential drought and heat stress; then, the plantlets were transferred to normal conditions (25 °C) for recovery. During the under-recovery phase, the leaves were collected to observe the stress state of the plants. In the leaves of *C. sinensis*, hydrogen peroxide was detected under drought conditions, and the brown precipitate was accumulated under sequential drought and heat stress. In the recovery phase, the hydrogen peroxide was almost removed at 120 min and undetectable at 240 min under the recovery conditions. In the leaves of *Q. acuta*, hydrogen peroxide was not detected under drought conditions, but it was observed from 90 min under drought–heat dual stress. In the recovery phase, hydrogen peroxide was almost removed at 120 min and undetectable after 480 min under recovery conditions. In the leaves of *Q. glauca*, hydrogen peroxide was induced under drought conditions. Under sequential drought and heat stress, it was clearly accumulated from 30 min. In the recovery phase, hydrogen peroxide was almost removed at 120 min and undetectable at 480 min under the recovery conditions. In the leaves of *Q. myrsinaefolia*, hydrogen peroxide was not detected under drought conditions, but it was shown from 30 min under sequential drought and heat stress, and it accumulated more with the exposure time. In the recovery phase, hydrogen peroxide was almost removed at 60 min and undetectable after 90 min under recovery conditions. In the leaves of *Q. phillyaeoides*, hydrogen peroxide began to be observed under drought conditions. Under sequential drought and heat stress, more hydrogen peroxide was accumulated. In the recovery phase, hydrogen peroxide was almost removed at 120 min and undetectable after 480 min under the recovery conditions. In the leaves of *T. japonica*, hydrogen peroxide was also observed under drought conditions. Under drought–heat dual stress, more hydrogen peroxide was accumulated. During the recovery phase, hydrogen peroxide was almost removed at 90 min and undetectable after 480 min under the recovery conditions. In the leaves of *A. julibrissin*, hydrogen peroxide was detected under drought conditions, and it accumulated more under sequential drought and heat stress. In the recovery phase, the hydrogen peroxide was not clearly removed until 480 min.

During the recovery phase following combined stress, the levels of H_2_O_2_ accumulation quantified via DAB staining exhibited diverse temporal patterns (Table 2). In the control group (A), all plant samples maintained low accumulation values (ranging from 0.2 to 0.3). However, in the H_2_O_2_-treated group (B), drought-treated group (C), and combined stress treatments (D–G), H_2_O_2_ levels increased significantly overall. Notably, following 60 min of combined stress (E), most treatment groups exhibited the highest levels of H_2_O_2_ accumulation (approximately 71–117), indicating that oxidative stress peaked due to heat-induced stimulation.

After transitioning to the recovery phase (H–N), some samples showed a relatively rapid decline in H_2_O_2_ concentration, whereas others maintained high levels of DAB staining even up to 480 min of recovery (N). For example, at time points J or L, transient increases in H_2_O_2_ were observed before decreasing again, suggesting that temporary ROS accumulation responses may be induced during recovery. Overall, a clear distinction was observed between species such as *C. sinensis* and *T. japonica*, which showed markedly reduced H_2_O_2_ levels during the later recovery stages (M–N), and species like *Q. acuta* and *A. julibrissin*, which continued to exhibit high accumulation. This indicates quantitative differences in recovery rates and detoxification efficiency among species.

## 4. Discussion

### 4.1. Establishment of Appropriate Sequential Drought and Heat Stress Conditions for Screening Stress-Tolerant Woody Plants

In this study, a screening protocol was established to evaluate tolerance to sequential drought and heat stress using 27 woody plant species growing in Korea, which were assessed based on physiological, phenotypic, and cellular responses under controlled environmental conditions. While many previous studies primarily focused on evaluating plant responses under single-stress conditions (either drought or heat), increasing attention has recently been directed toward the combined effects of sequential drought and heat stress due to intensifying climate change [10,28].

Our results demonstrated that *C. obtusa*, *Q. glauca*, and *Q. myrsinaefolia* exhibited 100% survival after exposure to two weeks of drought followed by heat stress at 45 °C. In contrast, *A. julibrissin* showed significantly reduced survival (8.3%) under the same conditions. These findings align with previous studies indicating that sequential drought and heat stress can cause more severe damage to plants than single stress alone, significantly threatening tree survival [1,4]. Although single heat stress treatments were not addressed here, they have been previously tested and reported. Our current findings show that plants subjected to combined drought and heat stress sustained more damage than those exposed to either stress individually. Indeed, sequential drought and heat stress are known to induce severe physiological disorders in woody plants, including photosynthesis inhibition due to water shortage, reduced growth, and excessive production of ROS that cause membrane and tissue damage, ultimately impairing plant survival [2,10]. However, the experiment was conducted for a short period using only one-year-old plants, which may not be sufficient for an accurate assessment of stress tolerance in woody species. Therefore, further studies should incorporate long-term monitoring of tolerant species to verify their resilience.

In this study, RWC and ELI were utilized as primary screening indicators. RWC is a representative indicator for evaluating water-retention capacity under drought conditions, as drought-tolerant plants generally maintain higher RWC levels [29]. Consistent with this, the sequential drought and heat stress-tolerant species identified in our experiment (*C. obtusa*, *Q. glauca*, and *Q. myrsinaefolia*) maintained high RWC values, indicating their strong capacity to retain water under drought stress conditions. Conversely, the sensitive species, *A. julibrissin*, showed significantly lower RWC values, verifying the effectiveness of this physiological parameter as an assessment criterion.

ELI, on the other hand, is widely used as a critical indicator of cell membrane stability under heat stress [30,31]. In this study, the identified tolerant species exhibited comparatively low ELI values under heat stress at 45 °C, reflecting less membrane damage and limited electrolyte leakage under elevated temperatures. Conversely, *A. julibrissin* displayed high ELI values, clearly corresponding to its significantly reduced survival rate and visible tissue damage under heat stress conditions. This reinforces the validity of ELI measurements for evaluating heat stress tolerance in plants.

Additionally, this physiological screening method was successfully used to identify several other woody species with strong tolerance to sequential drought and heat stress, including *C. sinensis*, *Q. acuta*, *Q. phillyraeoides*, *Q. salicina*, and *T. japonica*. These selected species, along with previously recognized tolerant species such as *Q. glauca* and *C. obtusa*, exhibited consistently high RWC and low ELI values, thus representing promising candidate species suitable for urban forestry, ecological restoration projects, and breeding programs aimed at mitigating climate change impacts. However, since the plant species were classified into only two groups (tolerant vs. sensitive) using a single stress condition, multiple stress conditions should be applied during tolerance assessment to further categorize species into more specific tolerance levels.

Although the present experiment was conducted with a limited number of three individuals per species, the results shown in Figure 2 indicate that the measured values of key physiological indicators, RWC and ELI, exhibited no substantial variation across species, suggesting that the reliability of the results is not significantly compromised. Therefore, this screening method can be considered a valid approach for the early selection of tolerant species under sequential drought and heat stress conditions. Nevertheless, considering the potential genetic and physiological variation within species, future studies should include a broader range of individuals to more precisely assess intra-species variability in stress tolerance.

Moreover, in this study, drought stress was applied prior to heat stress in a sequential manner, which could potentially act as drought priming to enhance tolerance to subsequent stress [32,33]. However, the results did not indicate any priming effects, possibly due to the absence of a recovery interval between the two stress phases. These observations emphasize the complexity of stress interactions in woody species and suggest that further investigation is required to better understand species-specific physiological responses to sequential stress events.

In addition, soil moisture content or water potential was not measured during the drought treatment. Although all plants were exposed to the same two-week irrigation withholding under controlled conditions, species-specific differences in transpiration or substrate retention may have caused variation in actual stress levels. Future studies should include soil moisture monitoring to ensure consistent drought severity across species and improve the accuracy of tolerance comparisons.

This study evaluated the stress tolerance of woody plants primarily through the physiological characteristics of the leaves. However, this approach may reflect short-term sensitivity rather than long-term tolerance. Specifically, it does not account for stress adaptations such as leaf shedding followed by rapid re-sprouting once favorable conditions resume observed in valonia oak [34]—traits that may suggest both sensitivity and resilience. Furthermore, individuals displaying minimal visible damage under stress may nonetheless experience reduced survival over time. These considerations underscore the complexity of stress responses in woody plants and highlight the importance of extended monitoring to accurately assess tolerance across species.

### 4.2. Phenotypic Responses of Candidate Woody Species Tolerant to Sequential Drought and Heat Stress

In this study, we analyzed the phenotypic characteristics of candidate woody species (*C. sinensis*, *Q. acuta*, *Q. phillyraeoides*, *Q. salicina*, and *T. japonica*) previously selected based on physiological assessments, including relative water content (RWC) and electrolyte leakage index (ELI), under sequential drought and heat stress conditions. Results showed that tolerant candidate species maintained predominantly green leaves, exhibited minimal leaf rolling, and showed negligible damage such as wilting or necrosis, even after two weeks of drought stress followed by exposure to 45 °C. Such stable phenotypes are typical indicators of high stress tolerance in woody plants, suggesting that these species possess strong physiological adaptability, minimal water loss under drought conditions, and high stability of cell membranes [1,35].

In particular, *Q. acuta*, *Q. phillyraeoides*, and *Q. salicina* exhibited excellent sequential drought and heat stress tolerance phenotypes. Although *T. japonica* showed slight leaf shrinkage overall, its phenotypic responses also indicated strong stress tolerance. These results align with previous studies demonstrating that deciduous broad-leaved trees, such as *Quercus* spp., exhibit robust physiological and morphological adaptations to sequential drought and heat stress [2,4].

In contrast, *A. julibrissin*, used as a sensitive control, displayed severe leaf wilting, discoloration, and rapid necrosis under identical stress conditions. These phenotypic responses corresponded closely with physiological indicators (RWC and ELI), confirming that phenotypic analysis is a valuable supplementary method for selecting stress-tolerant species. Previous studies have also reported rapid tissue damage and membrane disruption at early stages in sensitive species under stress conditions, supporting the reliability of the observed phenotypic responses [10].

The phenotypic results of this study confirm that candidate woody species selected through physiological assessments possess stable adaptation and high survival potential in real environmental conditions. Furthermore, combining physiological and phenotypic evaluations significantly enhances accuracy in assessing stress tolerance and selecting superior tree species. Therefore, these findings will serve as important baseline data for practical and effective woody plant resource selection in future urban forestry and ecological restoration projects.

### 4.3. Evaluation of Cellular-Level Tolerance to Sequential Drought and Heat Stress Using Evans Blue Staining Method

In this study, Evans blue staining was employed to precisely analyze cellular damage in woody plants selected for tolerant candidate species. Evans blue staining has been widely used for visually assessing cell membrane stability and effectively quantifying the proportion of dead cells within tissues under various stress conditions [30,36].

The selected tolerant candidate species, *C. sinensis*, *Q. acuta*, *Q. phillyraeoides*, *Q. salicina*, and *T. japonica*, showed very low cellular damage of approximately 15–30% or less, as determined by Evans blue staining, following two weeks of drought stress combined with high temperature exposure at 45 °C. These results indicate that these species possess strong cellular-level tolerance, maintaining membrane functionality and minimizing cellular damage even under severe sequential drought and heat stress conditions [30,37]. Particularly, the observed minimal cellular damage suggests that these plants effectively remove ROS and possess efficient antioxidant defense mechanisms, thereby maintaining cellular and tissue stability [28,38].

In contrast, the sensitive control species, *A. julibrissin*, exhibited severe cellular damage ranging from 45% to over 80% under identical stress conditions. These findings closely matched visual observations of wilting and necrosis, confirming earlier reports that sequential drought and heat stress induce rapid cell death and severe tissue necrosis in sensitive plants [30,39,40].

While previous studies have utilized Evans blue staining to evaluate plant tolerance under single stress conditions [41], research assessing cellular-level tolerance under simultaneous drought–heat dual stress has rarely been conducted. Moreover, this study demonstrated consistency between the cellular-level tolerance assessed by Evans blue staining and previously established physiological indices (RWC and ELI). Consequently, our findings validate the reliability and complementary nature of physiological, morphological, and cellular-level indicators in evaluating plant tolerance.

### 4.4. Assessment of Cellular Membrane Stability in Sequential Drought and Heat Stress-Tolerant Woody Plants Using Electrolyte Leakage Index (ELI)

In this study, the ELI was employed to quantitatively evaluate cellular membrane stability and damage levels in woody plants subjected to sequential drought and heat stress. ELI has been widely used as an effective physiological indicator for assessing plant tolerance to various abiotic stresses, especially heat stress [30,31]. High electrolyte leakage typically reflects increased cell membrane damage due to lipid peroxidation induced by excessive ROS production under severe abiotic stress conditions, ultimately leading to impaired cellular integrity and functionality [38].

The tolerant candidate species identified in this study—*C. sinensis*, *Q. acuta*, *Q. phillyraeoides*, *Q. salicina*, and *T. japonica*—consistently exhibited relatively low ELI values (below approximately 3.2) even after prolonged (two-week) drought followed by exposure to high temperature (45 °C). These results imply that these species possess inherent physiological mechanisms that effectively mitigate stress-induced membrane damage, maintaining cellular integrity and physiological function. Such characteristics may be attributed to efficient stress signaling, enhanced antioxidant capacity, or protective metabolites reducing oxidative damage under stress conditions [10,28,38].

In contrast, *A. julibrissin*, a stress-sensitive control, showed significantly elevated ELI values (10.0 after drought stress and 32.5 after sequential drought and heat stress). This increase clearly reflects severe cellular membrane damage and impaired recovery ability, consistent with previous findings where stress-sensitive plants showed increased electrolyte leakage due to substantial oxidative membrane damage under abiotic stress [30,42].

Notably, the results of ELI measurement correspond well to previous phenotypic observations and cellular damage assessments using Evans blue staining. These parallel findings suggest that ELI, along with morphological and histochemical methods, provides a robust and reliable assessment tool for evaluating plant tolerance under sequential drought and heat stress conditions. Furthermore, the fact that ELI values increased only slightly in tolerant species even under intensified stress indicates strong adaptive mechanisms that prevent extensive cellular damage, potentially including increased synthesis of antioxidant enzymes, osmotic adjustment, or enhanced membrane-protective compound accumulation [10,38].

In conclusion, the electrolyte leakage assay effectively differentiated sequential drought and heat stress woody plants from sensitive species, reinforcing its utility as a physiological screening criterion for selecting sequential drought and heat stress-tolerant woody plant species.

### 4.5. Recovery Capacity of Selected Woody Plants Under Sequential Drought and Heat Stress Based on DAB Staining Analysis

This study evaluated the recovery capacity of selected woody plant species after sequential drought and heat stress using 3,3′-diaminobenzidine (DAB) staining to visualize hydrogen peroxide (H_2_O_2_) accumulation. DAB staining is a widely used histochemical method for detecting H_2_O_2_ at the cellular level and provides insights into oxidative responses and antioxidant recovery mechanisms [26,38].

Quantitative image analysis was performed using Fiji (ImageJ), with a modified ROS analysis algorithm based on Sekulska-Nalewajko et al. [27]. Saturation values from DAB-stained images reflect H_2_O_2_ levels, where lower values indicate efficient detoxification. This approach enables objective, reproducible species comparisons beyond visual inspection. *T. japonica* showed a rapid decline in DAB values during recovery (120–480 min), suggesting robust antioxidant capacity and fast physiological recovery. Clustering and radar analysis confirmed *T. japonica* as the most resilient species.

By contrast, *A. julibrissin* maintained high saturation levels throughout recovery, indicating poor ROS scavenging and persistent oxidative damage—typical of stress-sensitive species [30,42].

Species such as *Q. acuta*, *Q. phillyraeoides*, and *C. sinensis* showed intermediate responses, with delayed but partial ROS reduction. These findings highlight interspecific variation in recovery timing and efficiency.

Notably, some species exhibited discrepancies between visual DAB intensity and quantified values. For instance, *A. julibrissin* showed intense staining despite low saturation values, likely due to differences in stain distribution or ROI selection. Improved image analysis methods are recommended for more robust assessments.

In summary, DAB staining combined with image-based quantification effectively revealed species-specific recovery capacity under abiotic stress. This method offers a valuable tool for evaluating plant stress tolerance and selecting resilient species for restoration and forest management under climate change [38,43].

### 4.6. Stepwise Physiological Responses to Sequential Drought and Heat Stress

Tolerant species maintained stable water status during the drought phase, exhibited limited oxidative damage under heat stress, and showed strong recovery capacity in the recovery phase (Table 3). In contrast, sensitive species experienced rapid physiological breakdown throughout the stress and recovery process. This progression presents a stepwise response model that provides practical criteria for selecting tolerant genotypes and guiding future research.

Nevertheless, this study was conducted over a short period using one-year-old seedlings, which may limit the accuracy of stress tolerance assessments across different woody species. Given that stress responses and recovery capacity in trees can vary depending on age and developmental stage, future studies should incorporate long-term monitoring to verify the persistence of tolerance and resilience traits.

Additionally, the transplantation process conducted prior to stress treatment may have introduced potential physiological stress, possibly influencing the observed responses. In this study, we minimized such effects by retaining the original soil around the roots during transplantation and allowing a 10-day acclimatization period before initiating the experiment. However, future studies should quantitatively assess and control for transplant-related variables to clarify their influence. Incorporating these considerations into experimental design will contribute to more accurate and ecologically relevant evaluations of dual stress tolerance in woody species.

## 5. Conclusions

**Figure 8:**
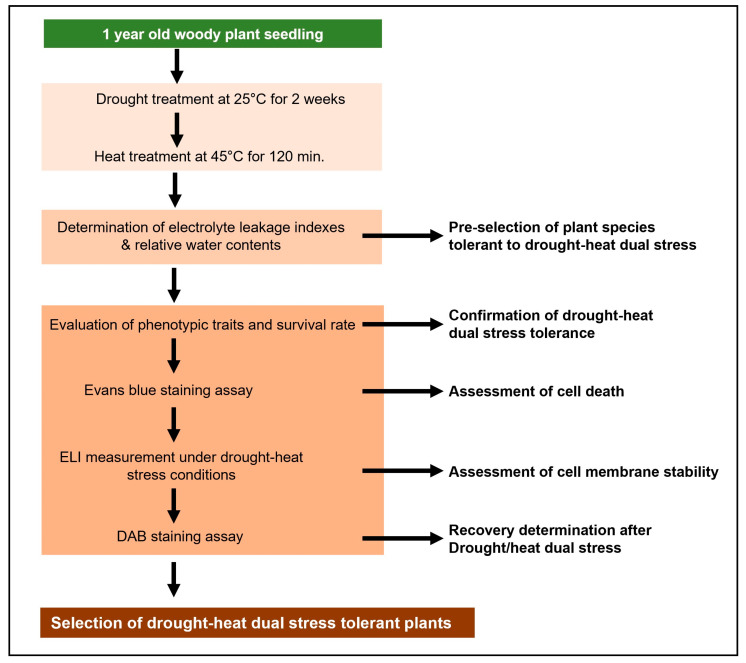
Screening strategy for drought–heat dual stress tolerance in woody plants.

This study successfully established a comprehensive and reliable screening platform to evaluate sequential drought and heat stress tolerance in 27 woody plant species native to Korea (Figure 8). To assess physiological responses under stress, RWC was used to evaluate water retention capacity, while ELI measured membrane damage through changes in permeability. Evans blue and DAB staining enabled visualization of cell death and oxidative stress, respectively. By integrating these histochemical indicators, we effectively identified stress-tolerant candidate species. Among the evaluated species, *C. sinensis*, *Q. acuta*, *Q. phillyraeoides*, *Q. salicina*, and *T. japonica* showed superior tolerance and recovery capacity, in addition to the previously recognized tolerant species *C. obtusa*, *Q. glauca*, and *Q. myrsinaefolia*. However, to more precisely understand the tolerance mechanisms of plants, it is necessary to design experiments using graded levels of sequential drought and heat stress. In particular, since plant responses to stress involve complex biochemical and molecular pathways, future research should include multifaceted analyses using biochemical markers such as antioxidant enzyme activity (e.g., SOD, CAT, POD), osmotic adjustment substances (e.g., proline, soluble sugars), as well as molecular-level investigations including gene expression analysis and the elucidation of stress-responsive signaling pathways. This study simulated sequential drought and heat stress conditions to evaluate the tolerance and recovery capacity of tree species, based on preliminary tests and previous studies. While the selected conditions were effective in distinguishing between tolerant and sensitive species, applying only a single stress intensity limited the ability to classify tolerance levels in detail. Future studies should apply various intensities and combinations of stress to enable more precise and quantitative assessments. In addition, this study proposed a stress tolerance assessment framework for woody seedlings; however, the short experimental period and potential transplantation-related variables may limit the interpretation. Future studies should incorporate long-term monitoring and strategies to control transplant stress for more robust evaluation. Nonetheless, the results of this study not only validate the robustness of the screening approach under combined drought and heat stress conditions but also provide a scientific foundation for the selection, breeding, and conservation of resilient tree species in response to ongoing climate change.

## Figures and Tables

**Table 1:** The list of woody plant species used in the study.

Scientific Name	Family	Common Name	Abbreviation
*Albizia julibrissin*	Fabaceae	Silk tree	Aj
*Amorpha fruticosa*	Fabaceae	False indigo bush	Af
*Camellia sinensis*	Theaceae	Tea camellia	Csin
*Castanopsis sieboldii*	Fagaceae	Siebold’s chinquapin	Csie
*Chamaecyparis obtusa*	Cupressaceae	Hinoki Cypress	Co
*Cinnamomum chekiangense*	Lauraceae	Japanese camphor tree	Cc
*Dendropanax trifidus*	Araliaceae	Korean dendropanax	Dt
*Eucommia ulmoides*	Eucommiaceae	Gutta percha	Eu
*Ilex cornuta*	Aquifoliaceae	Horned holly	Ico
*Ilex crenata*	Aquifoliaceae	Box-leaf holly	Icr
*Indigofera kirilowii*	Fabaceae	Kirilow’s indigo	Ik
*Indigofera pseudotinctoria*	Fabaceae	Dwarf false-indigo	Ip
*Lespedeza bicolor*	Fabaceae	Shrub lespedeza	Lb
*Lespedeza cyrtobotrya*	Fabaceae	Leafy lespedeza	Lc
*Ligustrum japonicum*	Oleaceae	Wax-leaf privet	Lj
*Morus bombycis*	Moraceae	Korean mulberry	Mb
*Neolitsea sericea*	Lauraceae	Sericeous newlitsea	Ns
*Pinus rigida*	Pinaceae	Pitch pine	Pr
*Quercus acuta*	Fagaceae	Red-wood evergreen oak	Qa
*Quercus glauca*	Fagaceae	Ring-cup oak	Qg
*Quercus myrsinaefolia*	Fagaceae	Bamboo-leaf oak	Qm
*Quercus phillyraeoides*	Fagaceae	Ubame oak	Qp
*Quercus salicina*	Fagaceae	Willow-leaf evergreen oak	Qs
*Robinia pseudoacacia*	Fabaceae	Black locust	Rp
*Sorbaria sorbifolia* var. *stellipila*	Rosaceae	False spiraea	Ss
*Spiraea prunifolia* f. *simpliciflora*	Rosaceae	Simple bridalwreath spiraea	Sp
*Ternstroemia japonica*	Pentaphylacaceae	Naked-anther ternstroemia	Tj

**Table 2:** Quantification of DAB staining intensity (mean ± SD), indicating H_2_O_2_ accumulation in six tree species across stress and recovery phases (A–N).

Species	A	B	C	D	E	F	G	H	I	J	K	L	M	N
*A. julibrissin*	0.2 ± 0.1 ^a^	18.4 ± 1.3 ^cd^	67.5 ± 3.4 ^b^	59.4 ± 3.1 ^d^	85.4 ± 4.2 ^b^	50.2 ± 2.6 ^d^	52.2 ± 3.2 ^d^	49.1 ± 2.3 ^e^	45.7 ± 2.2 ^e^	59.0 ± 2.9 ^e^	49.1 ± 2.5 ^e^	57.3 ± 2.7 ^d^	49.6 ± 2.4 ^c^	70.7 ± 3.6 ^c^
*C. sinensis*	0.2 ± 0.1 ^a^	23.6 ± 2.0 ^a^	83.3 ± 4.1 ^a^	92.8 ± 3.8 ^a^	116.6 ± 5.1 ^a^	47.9 ± 2.4 ^d^	41.9 ± 2.5 ^e^	70.2 ± 3.1 ^d^	63.2 ± 2.8 ^d^	52.9 ± 2.6 ^e^	52.4 ± 2.7 ^e^	49.4 ± 2.3	90.5 ± 3.9 ^b^	104.9 ± 4.4 ^b^
*Q. acuta*	0.1 ± 0.1 ^a^	21.9 ± 1.8 ^ab^	84.1 ± 3.6 ^a^	75.2 ± 4.0 ^b^	87.9 ± 4.7 ^b^	80.4 ± 3.9 ^b^	85.3 ± 4.4 ^b^	113.4 ± 4.9 ^b^	108.6 ± 4.5 ^b^	138.8 ± 5.3 ^b^	122.0 ± 4.9 ^b^	151.1 ± 5.8 ^a^	131.0 ± 5.5 ^a^	143.0 ± 5.4 ^a^
*Q. phillyraeoides*	0.3 ± 0.2 ^a^	22.9 ± 1.6 ^ab^	88.7 ± 4.2 ^a^	87.4 ± 3.7 ^a^	111.7 ± 5.2 ^a^	81.3 ± 4.0 ^b^	86.0 ± 4.1 ^b^	125.9 ± 5.4 ^a^	109.6 ± 4.8 ^b^	122.4 ± 5.1 ^c^	103.2 ± 4.2 ^c^	126.8 ± 5.0 ^c^	130.5 ± 5.3 ^a^	139.6 ± 5.5 ^a^
*Q. salicina*	0.2 ± 0.1 ^a^	16.4 ± 1.5 ^d^	57.3 ± 2.9 ^c^	74.2 ± 3.1 ^b^	114.3 ± 4.3 ^a^	95.0 ± 4.1 ^a^	98.9 ± 3.8 ^a^	132.2 ± 4.7 ^a^	126.0 ± 4.6 ^a^	150.1 ± 5.6 ^a^	129.0 ± 4.7 ^a^	136.3 ± 5.1 ^b^	134.3 ± 5.2 ^a^	137.9 ± 5.6 ^a^
*T. japonica*	0.3 ± 0.2 ^a^	20.5 ± 0.2 ^bc^	60.2 ± 0.2 ^c^	66.3 ± 0.2 ^c^	70.8 ± 0.2 ^c^	59.7 ± 0.2 ^c^	65.1 ± 0.2 ^c^	88.2 ± 0.2 ^c^	80.4 ± 0.2 ^c^	82.1 ± 0.2 ^d^	66.2 ± 0.2 ^d^	52.8 ± 0.2 ^de^	45.6 ± 0.2 ^c^	38.9 ± 0.2 ^d^

This quantification was conducted using DAB-stained leaf images (Figure 7), following a modified image analysis method proposed by Sekulska-Nalewajko et al. [27] and implemented in Fiji (ImageJ 1.53, NIH). In this approach, darker DAB staining corresponds to higher intensity values, as intense staining reduces light reflection. Accordingly, higher values indicate higher H_2_O_2_ accumulation, which reflects greater stress damage or slower recovery. Conversely, lower values indicate weaker staining, implying lower H_2_O_2_ levels and better stress tolerance or recovery. Based on this principle, DAB-stained images were analyzed to quantitatively assess H_2_O_2_ accumulation and recovery responses among tree species subjected to combined drought and heat stress. Data are expressed as mean ± standard deviation (SD) from three independent replicates (*n* = 3). Different letters (a–e) indicate statistically significant differences according to Duncan’s multiple range test at the *p* < 0.05 level.

**Table 3:** Stage-specific physiological responses of tolerant and sensitive woody species under sequential drought and heat stress.

Stage	Physiological Indicator	Response of Tolerant Species	Response of Sensitive Species
Drought	Relative water content (RWC)	High water retention (stable RWC)	Rapid RWC decline
Heat	Electrolyte leakage index (ELI)	Low membrane damage, low ROS accumulation	High electrolyte leakage, strong ROS signal
Recovery	Visual symptoms, DAB staining	ROS scavenging, tissue restoration	Persistent damage, mortality

## Data Availability

The original contributions presented in this study are included in the article. Further inquiries can be directed to the corresponding author.
